# Primary hepatic lymphoma a case report and literature review

**DOI:** 10.1097/MD.0000000000036688

**Published:** 2023-12-15

**Authors:** Minzhi Jiang, Shudian Jiang, Yu Yang, Rucheng Yao, Mingzheng Hu

**Affiliations:** a The First College of Clinical Medical Science, China Three Gorges University, Hubei Yichang, China; b Department of Hepatobiliary Surgery, Yichang Central People’s Hospital, Hubei, China.

**Keywords:** case report, liver tumors, lymphoma

## Abstract

**Rationale:**

Primary hepatic lymphoma is a rare extranodal non-Hodgkin lymphoma that is primarily localized in the liver. It predominantly affects elderly males and presents with nonspecific laboratory findings, imaging results, and clinical symptoms, making diagnosis challenging. Histopathological examination serves as the gold standard for diagnosis, and treatment options include chemotherapy or surgical intervention combined with chemotherapy.

**Patient concerns:**

A 50-year-old male patient came to our hospital for treatment after finding a mass in his liver.

**Diagnoses:**

Laboratory tests and clinical symptoms lack specificity for primary hepatic lymphoma, and imaging findings can be difficult to differentiate. Pathology is the gold standard.

**Outcomes:**

The patient was dead.

**Conclusion:**

A definitive diagnosis primarily relies on histopathological examination, and surgical resection combined with chemotherapy yields better treatment outcomes.

## 1. Introduction

Primary Hepatic Lymphoma (PHL) is a rare type of liver and non-Hodgkin lymphoma, which has no involvement in other tissues and organs and is common in elderly men. Without specific laboratory examination, imaging examination and clinical symptoms, he has great difficulty in diagnosis and pathological examination is the gold standard for diagnosis. Diffuse large B-cell lymphoma (DLBCL) is the most common and is treated with Rituximab, Cyclophosphamide, Doxorubicin, Vincristine, and Prednisone or surgical combination chemotherapy. The purpose of this paper is to explore the diagnosis and treatment of primary hepatic lymphoma, so as to improve the diagnosis and treatment efficiency of the disease.

## 2. Case report

### 2.1. Case background

Mr. Chen, a 50-year-old male, was referred to our outpatient clinic for further diagnosis and treatment. Three days prior, he had visited a local hospital where abdominal ultrasonography revealed a hypoechoic lesion in the left lobe of the liver, suggestive of an intrahepatic bile duct occupying lesion, as well as a hypoechoic lesion in the head of the pancreas also suggestive of an occupying lesion, and enlarged lymph nodes adjacent to the abdominal aorta. On physical examination, the patient had a flat and soft abdomen with no abdominal wall varices, tenderness, rebound tenderness, or abdominal masses. The liver and spleen were not palpable, and there were no signs of Murphy sign. There was no percussion tenderness in the liver area or renal area, and no shifting dullness was observed. Bowel sounds were normal, and there was no edema in the lower extremities. The patient had a history of smoking, was in good health, and denied any other medical conditions.

### 2.2. Laboratory and imaging examination findings

**Figure 1.**
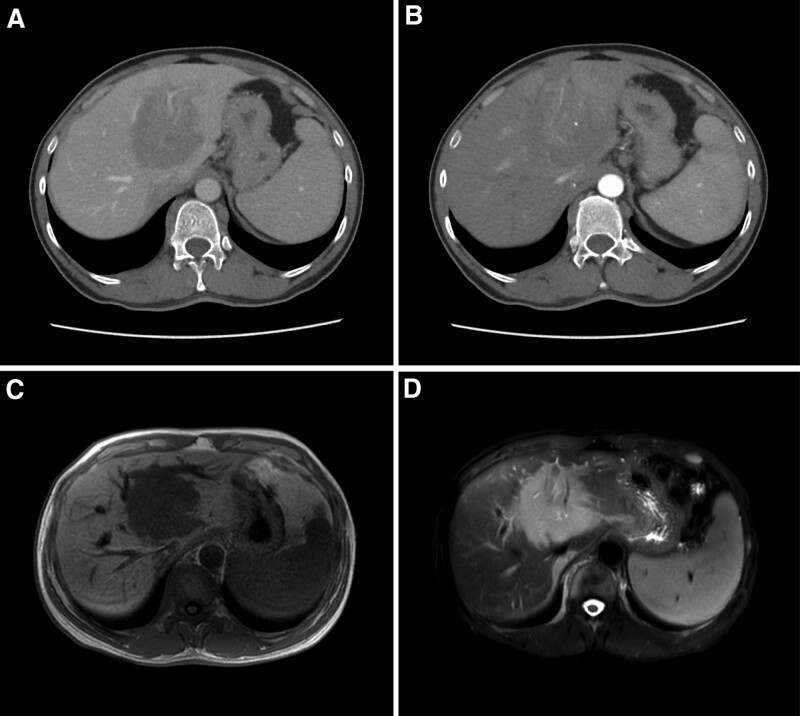
(A) Hepatic enhanced CT venous phase, in this image, a mass-like heterogeneous low-density shadow with indistinct borders is observed in the hepatic hilum region. (B) Hepatic enhanced CT arterial phase, the arterial phase CT scan reveals an irregular enhancement pattern within the liver. During the delayed phase, the degree of enhancement decreases. (C) Hepatobiliary pancreatic magnetic resonance cholangiopancreatography (MRCP) T1 phase, The T1 phase MRCP image exhibits a uniform low signal intensity throughout the liver. (D) Hepatobiliary pancreatic MRCP T2 phase, in the T2 phase MRCP image, a uniform high signal intensity is observed within the liver. CT = computed tomography.

On February 10, 2023, the patient underwent various laboratory and imaging examinations to assess their medical condition. The carbohydrate antigen 125 level was elevated at 118.0 U/mL. The levels of interleukin-2, interleukin-6, and interleukin-10 were measured at 7.88 pg/mL, 6.49 pg/mL, and 23.91 pg/mL, respectively. The prothrombin time was recorded at 20.2 seconds, the prothrombin activity at 44.0%, the international normalized ratio at 1.77, and the activated partial thromboplastin time at 59.4 seconds. It is essential to note that urine culture and identification showed no bacterial growth. The chest computed tomography (CT) scan results showed slightly enlarged axillary and mediastinal lymph nodes. An occupying lesion was observed in the hepatoduodenal region, along with increased and enlarged mesenteric and retroperitoneal lymph nodes. The abdominal CT scan revealed a high-density cyst in the left kidney. Similar to the chest CT scan findings, an occupying lesion was detected in the hepatoduodenal region, along with increased and enlarged mesenteric and retroperitoneal lymph nodes. Cystic lesions were observed in the liver, and calcification foci were found in the prostate. The liver gate-enhanced CT scan indicated the presence of a new lesion in the porta hepatis region, affecting the hepatic and common bile ducts, with signs of liver bile duct dilation. This finding raises suspicion of primary liver cancer. Increased and enlarged lymph nodes in the porta hepatis and retroperitoneal regions were noted, suggesting possible metastasis. The liver gate lesion seemed to receive blood supply from branches of the left and right hepatic arteries. Invasion of the main trunk and branches of the hepatic artery, the left branch of the portal vein, and the proximal portion of the middle hepatic vein were probable. Narrowing was identified in the intrahepatic segment of the inferior vena cava. Compression of the upper segments of both ureters was observed, causing hydronephrosis in the upper ureters and kidneys. Other findings include liver cysts and splenomegaly (Fig. 1A and B). The Liver-Gallbladder-Pancreas Magnetic Resonance Cholangiopancreatography scan confirmed the presence of an occupying lesion in the porta hepatis, indicating a potential new growth. Mild dilation of the intrahepatic bile ducts was observed. The porta hepatis and retroperitoneal lymph nodes showed increased size and partial fusion. Additional findings included liver cysts and dilation of the right renal pelvis and upper ureter. A small cyst was also identified in the right kidney (Fig. 1C and D). A mixed echogenic mass was observed in the left lateral lobe (segments S2–S3) of the liver, which suggests a high likelihood of hepatocellular carcinoma in ultrasound contrast imaging.

### 2.3. Pathological examination

**Figure 2.**
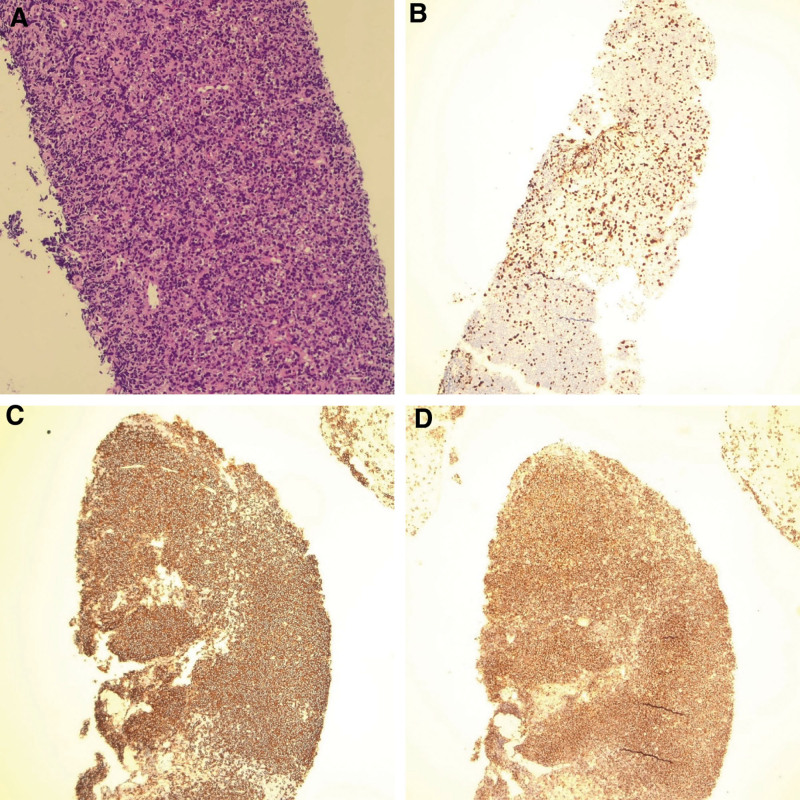
Histopathological results of liver mass biopsy. (A) Hematoxylin-eosin (HE) staining, magnified by 40 times. (B) Ki-67 antibody staining, magnified by 40 times. (C) Staining with CD20, magnified by 40 times. (D) Staining with BcL-2, magnified by 40 times.

On February 14, 2023, the patient underwent ultrasound-guided liver mass biopsy under local anesthesia. The pathological examination results (Fig. 2) revealed 2 liver tissue samples. Macroscopically, 2 gray-white linear structures were observed, measuring 1.7 cm and 1.6 cm in length, with a diameter of 0.1 cm each. Microscopically, diffuse sheets of atypical lymphocyte-like cells were observed in the liver tissue. Focally, a small amount of well-differentiated liver tissue was also seen. The lymphocyte-like cells showed a moderate enlargement, sparse cytoplasm, round to oval nuclei with slight irregularity, and distinct nucleoli. Immunohistochemistry results showed that the cells were negative for CD3 and CD21, positive for CD20, and had a Ki-67 labeling index of approximately 40%. The cells were negative for CD30, PCK, S-100, Cyclin D1, and SOX-11, weakly positive for Bcl-6 and MUM1, and positive for BcL-2, CD10, and CD5. Molecular pathology testing determined that the sample was negative for Epstein-Barr virus.

Based on the aforementioned findings, the diagnosis is consistent with non-Hodgkin B-cell lymphoma involving the liver tissue, most likely diffuse large B-cell lymphoma originating from a germinal center. This diagnosis is supported by the presence of atypical lymphocyte-like cells and immunohistochemistry results indicating the involvement of B-cell markers (CD20, CD10, BcL-2) and absence of T-cell markers (CD3). The Ki-67 labeling index of approximately 40% suggests a high proliferative activity of the neoplastic cells.

### 2.4. Clinical diagnosis and treatment

The patient presented with proteinuria and positive urinary occult blood, prompting consultation with relevant departments for comprehensive assessment and targeted management. Measures were taken to improve the patient’s coagulation function through symptomatic treatment. On the evening of February 16th, 2023, after the patient engaged in ambulation, they experienced dizziness, abdominal distension, abdominal pain, profuse sweating, generalized moist and cold skin, and progressively worsening symptoms. Considering the constellation of symptoms, physical signs, auxiliary examinations, and the presence of non-coagulating blood emanating from the lower right abdomen, the possibility of intra-abdominal bleeding was considered. Emergency fluid resuscitation was initiated, and DSA intervention was performed to achieve hemostasis. Meanwhile, symptomatic treatment was administered. However, despite these measures, the patient’s blood pressure continued to decline, and resuscitation efforts proved ineffective. Ultimately, at 23:41 on February 16th, 2023, the patient was declared clinically dead.

## 3. Discussion

### 3.1. Etiology

PHL is a rare type of non-Hodgkin Lymphoma that is limited to the liver without involvement of other organs. It accounts for <1% of all extranodal non-Hodgkin lymphomas, with secondary hepatic lymphoma being more common than primary lymphoma.^[1]^ PHL is often associated with hepatitis B virus or hepatitis C virus infections. Several studies have shown that individuals with concurrent hepatitis B virus/hepatitis C virus infections have a higher risk of developing PHL, possibly due to chronic inflammation leading to chronic proliferation of lymphocytes. However, the exact association between these infections and PHL is still a matter of debate.^[2–5]^ Furthermore, research suggests that the occurrence of PHL may be related to immune dysfunction.^[6–8]^ However, like the patient in this case, the etiology and specific triggers of PHL remain unknown, and its exact pathogenesis is still under investigation.

### 3.2. Clinical manifestations

PHL is more commonly seen in elderly individuals aged 50 to 70, with a male-to-female ratio of approximately 2:1.^[9,10]^ Most PHL patients do not present with specific symptoms, especially in the early stages. The condition is often incidentally detected during routine medical examinations when a liver mass is found. However, a small number of patients may experience abdominal pain, fever, jaundice, nausea, poor appetite, and night sweats.^[11–13]^ In the case of the patient described here, an elderly male, the liver mass was discovered during an evaluation for abnormal urination. There were no specific symptoms present. However, after seeking medical attention, the patient’s condition rapidly deteriorated, and a diagnostic puncture revealed non-coagulating blood. Based on comprehensive supplementary examinations, the possibility of tumor rupture and bleeding was considered more likely.

### 3.3. Diagnosis

#### 3.3.1. Laboratory investigation.

The most common finding in patients with PHL is an elevated level of lactate dehydrogenase (LDH), along with other abnormal liver function indicators. LDH is an enzyme found in various cells and tissues, including liver cells. Increased serum LDH levels may indicate tissue damage or cell death, which is commonly seen in cancer patients. Liver function indicators, such as aspartate transaminase (AST) and alanine transaminase (ALT), may also be elevated in some cases. These enzymes are released into the bloodstream when liver cells are damaged or undergo cell death. Therefore, assessing AST and ALT levels can provide valuable insights into liver function and assist in diagnosing PHL. Concurrent symptoms such as abdominal pain and fever may be accompanied by an increase in white blood cell count. Tumor markers associated with gastrointestinal malignancies, such as alpha-fetoprotein, carcinoembryonic antigen, and carbohydrate antigen, are usually within the normal range, as PHL primarily arises from lymphoid cells rather than epithelial cells. However, it is important to interpret these tumor markers cautiously, as their levels can be influenced by various factors and may not definitively rule out PHL. However, patients with multiple hepatic lesions should be considered for PHL diagnosis.^[14–16]^ Poor prognosis has been observed in patients with higher levels of carbohydrate antigen 125, AST/ALT, and LDH.^[17]^ In this particular case, the patient exhibited normal liver function indicators but showed abnormal coagulation function, which has not been reported previously. This could potentially be attributed to a deficiency or dysfunction of coagulation factors induced by PHL.

#### 3.3.2. Imaging examination.

PHL is generally detected through CT, magnetic resonance imaging (MRI), and other methods. Its imaging manifestations are difficult to distinguish from liver cancer and cholangiocarcinoma, especially when patients have liver cirrhosis.^[18]^ PHL typically presents as isolated or multiple masses, accounting for approximately 85% to 95% of cases. It can also show infiltrative growth, although this is less common. On non-enhanced CT scans, PHL appears as homogenous low-density, with the possibility of high-density indicating hemorrhage, and central low-density suggesting necrosis. Distinguishing it from primary liver cancer is challenging as primary liver cancer often exhibits changes in liver size, liver lobe proportions, and contour, along with the presence of liver cirrhosis and significantly elevated alpha-fetoprotein levels.^[19]^ In this case study, the patient’s non-enhanced CT showed a mass-like low-density shadow in the portal area. Although the alpha-fetoprotein level was within the normal range, differentiating it from primary liver cancer remained difficult, necessitating further liver-enhanced CT and MRI for diagnosis. On the enhanced CT scan, around 80% of PHL lesions showed no enhancement or minimal enhancement, with a few cases demonstrating calcifications. Distinguishing it from liver abscesses and primary liver cancer is challenging; liver abscesses can be assisted with ultrasound and symptoms such as chills and high fever, while primary liver cancer typically exhibits the characteristic “fast in, fast out” phenomenon in the arterial phase, with clear contrast between the lesion and surrounding liver parenchyma, and a clear peripheral low-density halo sign. However, when PHL involves the portal vein and its surroundings, a similar halo sign around the portal vein may appear, making differentiation more difficult. Additionally, in some patients, enhancement may occur in the periphery of the lesion while the central region does not enhance, resembling cholangiocarcinoma. Cholangiocarcinoma lesions can be present in the bile duct and show enhanced scans with sharp-edged linear water-like density. The delayed phase enhancement is a typical feature of cholangiocarcinoma. Elevated levels of carbohydrate antigen 199 and the appearance of symptoms such as jaundice further aid in the differential diagnosis.^[20,21]^ In this case study, the enhanced CT scan showed a poorly demarcated mass-like mixed low-density shadow with uneven enhancement in the arterial phase and decreased enhancement in the delayed phase, still making differentiation from primary liver cancer difficult. In MRI on PHL, the majority of cases show uniform low signal or iso-signal on T1-weighted images and high signal on T2-weighted images. Occasionally, it may also show a low signal on T2-weighted images with enhancement in the peripheral region, which is similar to the findings on enhanced MRI. This makes differentiation from primary liver cancer and cholangiocarcinoma particularly challenging.^[20,22,23]^ In this case study, the MRI showed a mass-like shadow with a high signal on T2-weighted imaging. Considering the comprehensive examination results, the initial consideration was primary liver cancer. To establish a definitive diagnosis and formulate a treatment plan, the patient underwent recommended lesion tissue biopsy. Radiologists and clinicians must collaborate closely to ensure a cmprehensive and multidisciplinary approach in the evaluation and treatment of patients with hepatic abnormalities.

#### 3.3.3. Pathological examination.

DLBCL, follicular lymphoma, T-cell lymphoma, and mucosa-associated lymphoid tissue lymphoma are commonly seen types of PHL, with DLBCL and mucosa-associated lymphoid tissue being the most frequently reported ones.^[24]^ Macroscopically, PHL is often observed as irregularly bordered, firm masses measuring 2-9cm, with a fish-like appearance on cross-section and occasional focal necrosis.^[25]^ The tumor cells in DLBCL typically exhibit diffuse enlargement and varied lymphoid morphology, expressing B-cell markers CD19, CD20, CD22, PAX5, and CD79a. Based on gene expression profiles, DLBCL can be further classified into germinal center B-cell-like subtype, activated B-cell-like subtype, and primary mediastinal (thymic) large B-cell lymphoma, with B-cell-like subtype often showing positivity for BCL-6 and CD10.^[26]^ In this case, the patient presented with the most common type of DLBCL, measuring approximately 8*7*7 cm. Microscopically, diffuse sheets of medium-sized to large atypical lymphoid cells were observed, with immunohistochemical staining for CD3 (−), CD20 (+), BcL-2 (+), and weak Bcl-6 expression, confirming the diagnosis of DLBCL.

### 3.4. Treatment and prognosis

PHL can undergo chemotherapy or surgery depending on their specific circumstances. Similar to other non-Hodgkin lymphomas, the rituximab, cyclophosphamide, doxorubicin, vincristine, and prednisone regimen is the preferred choice, with a success rate of achieving disease control in 60% of DLBCL patients. Additionally, for those whose immunohistochemistry indicates CD20 (+), the addition of the CD20-targeted drug rituximab is recommended. However, further research is necessary to improve the efficacy of this treatment approach.^[27]^ Moreover, based on current research, the overall survival period for PHL patients following surgical intervention can extend up to 10 years. Nevertheless, only a small number of patients are candidates for surgical treatment, and the effectiveness of surgery remains unclear. PHL patients who undergo liver resection exhibit better survival rates, which may be attributed to both the surgery itself and the correlation between tumor status and overall health. These findings suggest that a combination of surgical resection and chemotherapy (either before or after surgery) can provide better treatment outcomes for patients. However, due to the limited number of cases, further studies are required to validate these findings.^[28]^

## 4. Conclusion

PHL is a rare extranodal lymphoma that primarily originates in the liver. Its diagnostic challenges stem from the lack of specificity in laboratory tests and clinical symptoms, particularly in distinguishing it from hepatocellular carcinoma and cholangiocarcinoma. Improving the level of imaging examinations can aid in auxiliary diagnosis, but definitive diagnosis still relies on pathology. The CHOP chemotherapy regimen serves as the gold standard, and if feasible, liver resection is preferred. Further research is needed to enhance our diagnostic capabilities and treatment efficiency.

## Acknowledgments

Thanks to my my teachers and my friends.

## Author contributions

**Conceptualization:** Mingzheng Hu.

**Resources:** Mingzheng Hu.

**Supervision:** Mingzheng Hu.

**Writing – original draft:** Minzhi Jiang, Shudian Jiang, Yu Yang, Rucheng Yao.
